# Seasonal PM 2.5 exposures induce differential responses to influenza A virus infection in primary human airway epithelial cells

**DOI:** 10.21203/rs.3.rs-6890544/v1

**Published:** 2025-07-10

**Authors:** Stephanie A Brocke, Timothy Smyth, Hong Dang, Adam Speen, Yong Ho Kim, Cara Christianson, Kasey Kovalcik, Joseph Patrick Pancras, Michael Hays, Zhen An, Weidong Wu, Ilona Jaspers

**Affiliations:** University of North Carolina; University of North Carolina; University of North Carolina; University of North Carolina; United States Environmental Protection Agency; United States Environmental Protection Agency; United States Environmental Protection Agency; United States Environmental Protection Agency; United States Environmental Protection Agency; Xinxiang Medical University; Xinxiang Medical University; University of North Carolina

## Abstract

**Background:**

Air pollution, specifically fine particulate matter (PM_2.5_), in China is responsible for millions of excess deaths each decade. Examinations of Chinese municipalities have revealed correlations between ambient PM_2.5_ levels and the prevalence and severity of respiratory viral infections. Seasonal sources of ambient PM_2.5_ vary, with coal combustion for indoor heating significantly contributing during colder months. Due to this seasonality, we sought to investigate whether exposure to seasonal PM_2.5_ collected in Xinxiang, China would differentially alter the response to subsequent influenza A/California/04/2009 (H1N1) viral infection in a primary human nasal epithelial cell (HNEC) culture model. After the PM_2.5_ samples were chemically analyzed, HNECs collected from males (N = 4) and females (N = 3) grown at air-liquid interface were exposed to 22 μg/cm^2^ of seasonal PM_2.5_ followed by inoculation with influenza A H1N1 at MOI = 0.001. At 2 and 24 h post infection (p.i.) we assessed transcriptional changes and basolateral release of immune and antiviral mediators.

**Results:**

Summer and fall PM_2.5_ samples contained a greater organic carbon mass fraction compared to winter and spring. Winter contained the largest mass fraction of anionic components and spring the largest inorganic element mass fraction. In response to infection alone without PM_2.5_ exposure, the transcriptional response to infection at 24 h p.i. differed between the sexes with males having more robust interferon pathway activation. Exposure to the seasonal PM_2.5_ samples without infection induced a moderate transcriptional response at 2 h, with the winter PM_2.5_ inducing the greatest response. The seasonal PM_2.5_ exposures followed by viral infection resulted in a more robust transcriptional response at 2 h p.i. with the winter, spring, and fall PM_2.5_ samples (but not the summer PM_2.5_) upregulating many inflammatory pathways. At 24 h p.i., only the spring PM_2.5_ sample increased inflammatory and antiviral mediator proteins in the basolateral medium, while winter PM_2.5_ increased these inflammatory markers in the mock infected cultures.

**Conclusions:**

Chemical differences in seasonal PM_2.5_ from the winter, spring, and fall, coinciding with influenza season, likely contribute to the adjuvant pro-inflammatory effects of exposure on antiviral host response. Heightened inflammation early in infection could contribute to worsened pathogenesis.

## Background

Air pollution remains a critical public health concern in China. In 2019, the country lost an estimated 1.85 million lives due to air pollution exposure, an increase from 1.24 million air pollution-related deaths in 2017^1–2^. Stringent emissions regulations adopted by the Chinese government have resulted in a steady decline in average annual fine particulate matter (PM_2.5_) concentrations from 2013–2022^3–4^. However, the 2023 World Air Quality Report showed a reversal of this trend in 2023, with the first annual PM_2.5_ concentration increase in 10 years since the enaction of China’s Air Pollution Action Plan^5^. Despite improvements in air quality, 0.2% of cities in East Asia met the World Health Organization’s annual PM_2.5_ guideline in 2023, compared to 14% of North American cities^5^.

In the past 10 years, a number of studies have examined the association between ambient PM_2.5_ and rates of influenza, influenza-like illness, or acute respiratory tract infections in individual Chinese cities^6–13^. Overwhelmingly, these studies have found a positive association between ambient PM_2.5_ levels and case numbers, though they disagree in the reported lag time for this effect. Furthermore, studies examining many cities across multiple Chinese provinces have reported the same conclusions associating increases in PM_2.5_ with increased cases of influenza^14–16^ and COVID-19^17^.

In the World Air Quality Report in 2023, Xinxiang ranked 13th among Chinese cities with the highest air pollution^5^. Experimental approaches using ambient PM_2.5_ collected in Xinxiang indicate that exposure alters response to viral infection in the airway epithelium^18–19^. Exposing a bronchial epithelial cell line to PM_2.5_ (collected from December 2020 to March 2021) prior to influenza H3N2 infection resulted in increased pro-inflammatory cytokine release, increased susceptibility to infection, and differential transcriptomic changes in response to infection^18–19^.

Ambient PM_2.5_ fluctuates in concentration and chemical composition seasonally^20–23^ due to changes in meteorological conditions and anthropogenic activity. In China, winter heating fueled by coal combustion contributes to 18% of annual average PM_2.5_^23^ or more^24^, and significantly worsens the air quality index^25^. The increase in PM_2.5_ due to solid fuel combustion in colder months has been linked to increased mortality^25^. Collection and chemical analysis of seasonal PM_2.5_ in Beijing from spring 2009 to winter 2010 revealed that sources of PM_2.5_ vary significantly between the seasons; biomass burning and soil dust contribute much more to PM_2.5_ in spring and autumn while secondary organic aerosols contribute most in summer^23^. Furthermore, in Beijing there was an association between PM_2.5_ and influenza-like illness within flu season (October to April), but no association from May to September, even after controlling for temperature. In terms of experimental approaches, the connection between seasonal PM composition and biological outcomes in the context of viral infection remains understudied.

In the present study, we sought to assess differences in seasonal PM chemistry and how they impact response to influenza H1N1 infection in primary human nasal epithelial cells (HNECs) grown at air-liquid interface. This organotypic model system allows for the investigation of confounders such as biological sex and inter-individual genetic differences on the effects of PM exposure, influenza infection, and their interaction. Ambient PM_2.5_ was collected over a yearlong period in Xinxiang from November 2021 to October 2022. The organic carbon, elemental carbon, inorganic elements, and anionic components were measured in the samples. In HNECs from 7 donors (3F, 4M), we then evaluated effects of four seasonal PM samples, influenza H1N1 infection, and their interaction on transcriptomic changes at 2 and 24 h post infection and expression of cytokines and chemokines at 24 h post infection. We found that exposure to seasonal PM samples induced differential responses, namely upregulation of pro-inflammatory signaling pathways and mediators among infected cells compared to unexposed controls. We hypothesize that chemical differences in PM from different seasons drive the differential responses they induce at the transcript and protein levels.

## Method

### Particulate Matter Collection and Extraction

Ambient PM_2.5_ was sampled on 36 separate days atop the roof of a research building on the Xinxiang Medical University campus (35◦17′8.67′’N, 113◦55′37.87′’E) in Xinxiang, Henan Province, China. Sampling occurred for continuous 4 or 8 h daytime periods between November 2021 and October 2022. The two main roads nearest the sampling site were 120 m West and 150 m North. Sampling was conducted using a high-volume impactor (TE-6070VFC-PM2.5, Tisch Environmental, Cleves, OH USA) with a size cut at 2.5 μm and a 1.13 m^3^/min flow rate. The impactor was calibrated per the manufacturer’s instructions monthly. Daily PM_2.5_ was collected onto polytetrafluoroethylene (PTFE) filter sheets (PF-TB5W-S, Cobetter Filtration Equipment CO., LTD, Hangzhou, Zhejiang, China). Filters were stored at −20°C until extraction. For PM extraction, filters were divided into quarters and pieces were submerged in 90% v/v HPLC-grade methanol:10% ultrapure water and sonicated in an ultrasonic Emerson Branson water bath (Emerson Electric Co., St. Louis, MO, USA) at 40 kHz for 15 minutes. After sonication, the extracts were passed through 40 μm sieves before being dried down under a stream of ultra-high purity N_2_ gas in 50 ml polypropylene tubes. Once the remaining volume was < 1.7 ml, the PM slurry was transferred to pre-weighed 1.7 ml polypropylene tubes and the samples were fully dried under N_2_. Once dry, the tubes were stored open in a covered desiccator overnight. The tubes were again weighed, and PM mass of each sample was calculated. Method blanks using blank filter material were also generated according to the same protocol. For chemical analysis, samples were resuspended in 90% v/v HPLC-grade methanol in ultrapure water and again sonicated at 40 kHz for 5 minutes. The resuspended samples were serially diluted to a final concentration of 1 mg/ml. Three aliquots of each sample containing 100 μg PM each were added to pre-cleaned 15 ml polypropylene conical tubes and submitted for OC/EC, ICP-MS, and IC analyses at the US Environmental Protection Agency (Research Triangle Park, NC, USA).

### Organic/elemental carbon (OC/EC) composition analysis of PM

Samples were analyzed for OC/EC composition using a Sunset Laboratory Model 5L Thermal-Optical Carbon Analyzer (Sunset Laboratory Inc., Tigard, OR). In preparation for analysis, each sample solution was homogenized using a vortex mixer for one minute. An aliquot of 20 μl was extracted from the well-mixed solution using a syringe and directly spiked onto the center of a clean quartz filter punch. The spiked filter punch was dried using the equipment’s drying cycle function to ensure methanol evaporation. Once dried, the OC/EC analysis was conducted using NIOSH 870 parameters^26^ (NIOSH Method 5040). The resulting organic carbon, elemental carbon, and total carbon values were recorded.

### Inorganic element analysis of PM

To determine inorganic elemental concentrations in the filter PM, an aliquot of the PM extract containing 100 μg of PM was transferred into an acid-cleaned SCP Science digiTube. Then, 0.5 ml of HNO_3_ – HCl (3:1) mixture was added, capped, and placed on an SCP DigiPREP Jr block digester at 65°C for 3 h. The next day, 9.5 ml of 18.2 mΩ water was added into each tube and vortex mixed. Elemental concentrations were measured using a Thermo Finnigan Element2 (Bremen, Germany) sector field inductively coupled plasma mass spectrometer (HR-ICPMS) housed in a class 100 clean laboratory at the EPA facility in Research Triangle Park, NC. External calibrations were performed with custom multi-element standards from High Purity Standards (Charleston, SC, USA). Calibration standard curves were deemed acceptable if regression (r^2^) values were greater than 0.99. An internal standard (2 ppb Indium) solution was introduced in-line along with samples to account for analytical signal drift. National Institute of Standards and Technology-certified standard reference materials SRM 1640 and SRM 1643 were used to verify instrument performance and analytical accuracy.

### Ion chromatography analysis of PM

Samples were diluted to 5 ml using Milli-Q water (Millipore Sigma, MA, USA) prior to transfer into Thermo Scientific Dionex vials and Guardcaps. Ion Chromatography analysis was performed on a Dionex ICS-2000 (Chromeleon software v6.8) equipped with a 200 μl injection loop, Thermo Scientific Dionex IonPac AS18 anion-exchange analytical (4×250mm, pn060549) and AG18 guard columns (4×50mm, pn060551) utilizing a 30 mM potassium hydroxide (KOH) isocratic separation method, based on EPA Method 300.0(A)^27^. The system was calibrated using a Thermo Seven Anion standard (pn056933) of fluoride, chloride, nitrite, bromide, sulfate, nitrate and phosphate. Each sample was injected and analyzed twice to observe precision.

### Chemical composition data analysis

Because a portion of all PM filters was reserved and not extracted, the PM mass collected from the extracted portion was divided by the fraction extracted to extrapolate the total mass of PM on the entire filter. Concentrations of analytes were provided in μg/g of PM. Ambient air concentrations of analytes were determined by multiplying μg/g by the extrapolated total g of PM obtained per sample and dividing by the total volume of air sampled. For the OC/EC data, samples were corrected with the reagent blank (90% v/v HPLC-grade methanol in ultrapure water). The ICP-MS and IC data were corrected with the filter blank data. Other data visualization was performed in GraphPad Prism software (v. 10.2.0).

### Culture of Human Nasal Epithelial Cells (HNECs)

Human nasal epithelial cells from n = 7 healthy adult donors (3F, 4M) were obtained by a University of North Carolina Institutional Review Board-approved protocol (IRB 11–1363), and written informed consent was obtained from all donors. Demographic information on HNEC donors is provided in Table 1. Details of culturing methods are as previously described^28–29^. Briefly, cells were expanded in flasks using PneumaCult Expansion Plus medium (STEMCELL Technologies, Vancouver, BC, Canada) for three passages before being seeded on CellTreat brand (Pepperell, MA, USA) permeable culture inserts. Upon confluency, the apical medium was removed and basolateral medium switched to PneumaCult ALI medium. The cells differentiated at the air-liquid interface (ALI) for 4 weeks until mucus production and motile cilia were observed in all cultures with medium changes and apical washes three times per week.

### Exposure to seasonal PM and infection with Influenza A

Four PM_2.5_ samples, collected in approximately 3-month intervals over one year were selected for exposures to be followed by influenza A/California/04/2009 (H1N1) virus infection. Further details about preparation of viral inoculum are provided in the Supplemental Methods. The samples selected were collected on; December 11, 2021 (referred to as Winter PM); March 18, 2022 (Spring PM); June 11, 2022 (Summer PM); and September 28, 2022 (Fall PM). Dried PM samples were resuspended in pure water before dilution in ALI culture medium for exposure. Well-differentiated HNECs at ALI from 7 donors (3F, 4M) were exposed to PM from Winter, Spring, and Summer at 22 μg/cm^2^ for 2 h. Due to limitations with available cultures, only 4 of 7 donors were exposed to Fall PM (2F, 2M). At the end of the 2 h exposure period, apical exposures were carefully removed and 10 μl of influenza A inoculum at a multiplicity of infection (MOI) of 0.001 (or mock) was applied to the apical surface of cultures. At 2, 24, and 48 h post infection, basolateral supernatant and cell lysates were collected.

### RNA sample processing and RNA Sequencing

RNA was purified from cell lysates using a commercially available kit (PureLink RNA Mini Kit, Thermo Fisher Scientific, Waltham, MA, USA). Samples which would be directly compared were extracted in the same batch to avoid batch effects. Purified RNA from the 2 and 24 h timepoints were submitted to Genewiz (Azenta Life Sciences, Burlington, MA, USA) for Standard RNA sequencing services. RNA concentration and integrity were checked prior to library preparation. RNA sequencing libraries were prepared using the NEBNext Ultra II RNA Library Prep Kit for Illumina per the manufacturer’s instructions. The sequencing library was validated on the Agilent TapeStation (Agilent Technologies, Palo Alto, CA, USA) and quantified by Qubit 2.0 Fluorometer (Invitrogen, Carlsbad, CA, USA) as well as by quantitative PCR (KAPA Biosystems, Wilmington, MA, USA). Sequencing libraries were then multiplexed and clustered onto a flowcell on the Illumina NovaSeq instrument according to manufacturer instructions. The samples were sequenced using a 2×150bp Paired End configuration. Image analysis and base calling were conducted by the NovaSeq Control Software. Raw sequence data generated from Illumina NovaSeq was converted into fastq files and de-multiplexed using Illumina bcl2fastq software (v. 2.20). One mis-match was allowed for index sequence identification.

### RNA Sequencing Data Analysis

Paired-end reads were first evaluated for quality using FastQC^30^ (v.0.12.1). The GENCODE *Homo sapiens* genome and annotation files (Release 45, GRCh38.p14) were used as the reference for human gene expression. Additionally, the NCBI RefSeq genome of Influenza virus A/California/07/2009 (H1N1) (GCF_001343785.1) was used as a reference for viral genes. To simultaneously evaluate viral gene expression in infected host cells, the viral genome and annotation files were concatenated onto the respective human files. Reads were aligned and mapped to the reference using STAR^31^ (v.2.7.11a). Binary alignment map files were indexed using SAMtools^32^ (v.1.19). Lastly Stringtie2^33^ (v.2.1.5) was used for transcript assembly. To ensure read counts of human genes were normalized between the two infection groups, transcript assembly was repeated using only the human reference genome to generate human-only gene count matrices.

To accommodate donor as a random effect in differential expression analysis, Dream^34^ functions within the Bioconductor variancePartition package were used. Any genes with summed raw counts fewer than 100 across all samples were removed from further analyses. A full model was fit with infection (mock or virus), exposure (Control, Winter, Spring, Summer, Fall), and timepoint (2 or 24 h) as fixed effects and donor as a random effect. Subsequently, subgroup analyses between levels of variables were performed for specific contrasts of interest. Volcano plots, Euler diagrams, and heatmaps were generated using differential expression data using the ggplot2, eulerr, and ComplexHeatmap packages with cutoffs of |fold change ratio| >1.5 and q value < 0.1. Word clouds presenting major biological terms predicted to be impacted by differentially expressed genes present within indicated groups were generated using Genes2WordCloud^35^. Gene lists were assembled based on overlapping or exposure group-specific differential expression patterns. Common English words and common biological terms were removed while a frequency significance value of 2.5 was employed.

### Measurement of protein biomarker concentrations

Basolateral supernatants collected 24 and 48 h post infection were analyzed for biomarkers implicated in response to viral infection. Kits were purchased from MesoScale Discovery (Rockville, MD, USA) and BD Biosciences (Franklin Lakes, NJ, USA) and assays were performed according to manufacturer instructions. Analytes included IFN-α2a, IFN-β, IFN-γ, IFN-λ1, IL-1β, IL-4, IL-5, IL-6, IL-7, IL-8, IL-9, IL-10, IL-12p70, TNF-α, G-CSF, GM-CSF, IP-10, MCP-1, MIP-1α, and VEGF-A. Missing values as a result of levels below detection limit were replaced with half the minimum detected concentration in the sample set.

### Protein biomarker data analysis

Analysis and visualization of the timepoint-stratified biomarker concentration data were performed in GraphPad Prism software (v.10.2.0) by a mixed-effects model with infection (mock or virus) and exposure (Control, Winter, Spring, Summer, Fall) as fixed effects and donor as a random effect. P values ≤ 0.05 were considered significant. Dunnett’s post hoc test was used to test for differences between groups, * P ≤ 0.05, ** P ≤ 0.01, *** P ≤ 0.001.

## Results

### Differential expression analysis revealed interactions between infection status and collection timepoint

To evaluate the effects of H1N1 infection, PM exposure, and donor sex on the transcriptome of HNEC samples from n = 7 donors, RNA purified from cell lysates at 2 and 24 h post infection was sequenced. A linear mixed model incorporating sample donor, collection timepoint, infection status, PM exposure status, and donor sex revealed that interindividual transcriptomic differences was the greatest driver of sample variability while donor sex contributed a negligible impact on variance (Supplemental Figure S1A). Regardless, following incorporation of donor as a random effect in our mixed effects model (See Methods), differential expression (DE) analysis differentiated by donor sex was assessed. As expected, DE analysis demonstrated the majority of differentially expressed genes at the 2 and 24 h timepoint were Y- and X-chromosome linked, with only two autosomal genes, *ADH4* and *ENPP2*, demonstrating differential expression at either timepoint (Supplemental Figure S1B/C). Further, there were no statistically significant differences in normalized counts of influenza H1N1 viral genes between the sexes at 2 or 24 hours (Supplemental Figure S1D/1E). As such, further analysis was conducted accounting for sample donor without incorporating sample donor sex in the mixed effects model.

**Figure 1:**
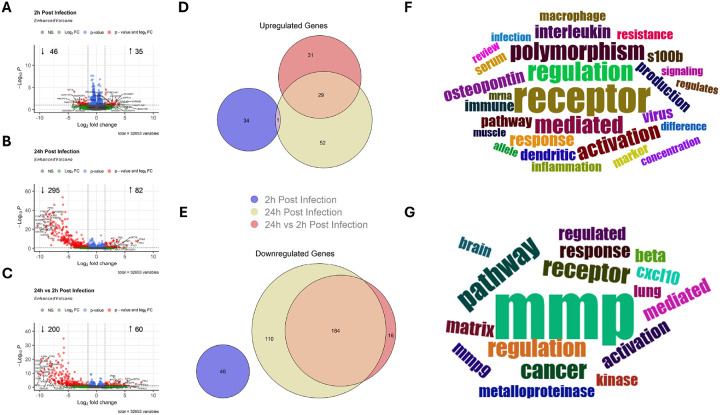
Virally Infected Human Nasal Epithelial Cells Demonstrate Distinct Early and Late Phase Gene Expression Patterns. Volcano plots demonstrating significantly differentially expressed genes (DEGs) compared to mock infected samples at A) 2 hours post infection or B) 24 hours post infection or C) 24 hours post infection compared to 2 hours post infection. The top 10 DEGs by positive and negative log_2_ fold change and total number of up- and down-regulated genes are presented. D-E) Euler plots presenting the number of unique and overlapping DEGs between treatment groups. Word clouds demonstrating major biological themes impacted by DEGs F) shared between or G) unique to 24 hour post infection samples compared to mock infected and/or 2 hour post infection samples.

Viral infection induced changes in gene expression at 2 and 24 h p.i. relative to mock infection, however, only a small subset of DE genes was detected at 2 h p.i. (Fig. 1A–B). Further, differential expression of 24 h p.i. relative to 2 h p.i. demonstrated a large number of significantly differentially expressed genes (Fig. 1C). Importantly, the majority of DE genes detected between virally infected samples at 24 h p.i. relative to 2 h p.i. were similarly detected between samples 24 h p.i. relative to mock infection (Fig. 1D/1E). Only one DE gene, a long non-coding RNA (lncRNA), overlapped at 2 and 24 h p.i. relative to mock infection (Fig. 1D, Supplemental File SF1), demonstrating distinct responses to influenza infection between the very early and late infection response.

Further investigation of major themes represented by DEGs shared between samples 24 h p.i. relative to mock infection versus 24 h p.i. relative to 2 h p.i. suggest a large degree of modification of cell surface receptor expression (Fig. 1F) including cluster of differentiation (CD), C-type lectin, FC gamma receptor, and G protein-coupled receptor (GCPR) genes (Supplemental File SF1). To a lesser extent, modification of interleukin-linked genes, immune activity, and inflammation was observed (Fig. 1F) in large part due to modifications of complement, interleukins, interferons, and interferon inducible genes (Supplemental File SF1). In contrast, genes which were unique to a single group demonstrated the greatest activity linked to matrix metalloproteinases (MMPs) as demonstrated by enrichment of the terms mmp, mmp9, metalloproteinases, and matrix (Fig. 1G) driven by *MMP9* and *ADAMTS4* downregulation in 24 h p.i. samples compared to mock infection and *ADAMTS1* upregulation in 24 h p.i. relative to 2 h p.i. (Supplemental Files SF1). However, in agreement with Fig. 1F, modification of cell surface receptors was also enriched (Fig. 1G) driven by several CD genes, FC gamma receptors, and solute carrier family genes (Supplemental File SF1). Importantly, despite 183 DEGs demonstrating significant differential expression in a single group, similar directionality patterns were observed between 24 h p.i. regardless of reference group while opposite trends were observed between 2 h p.i. samples and either 24 h p.i. comparison (Supplemental File SF1). Further, only one gene, *PILRA*, demonstrated significant differential expression in each group, however, 2 h p.i. samples demonstrated significant upregulation while both 24 h p.i. groups demonstrated significant downregulation (Supplemental File SF1).

#### Seasonal differences in chemical composition of PM_2.5_ from Xinxiang.

The mass of PM extracted from filters was used to determine ambient PM_2.5_ concentrations at the sampling site in Xinxiang, presented in Supplemental Figure S2A. Generally, ambient PM concentrations were highest between November 2021 through March 2022, and remained relatively low from May to October of 2022. The correlation between this extrapolated PM_2.5_ concentration and reported PM_2.5_ concentration data from a nearby Xinxiang air quality monitoring station, publicly available online (aqicn.org/city/xinxiang), was determined to be R^2^ = 0.729, shown in Supplemental Figure S2B. Organic carbon is lowest in the late fall-late winter months, from November 2021 to March 2022, and is generally higher from spring through middle of fall of 2022 (Supplemental Figure S2C). Spring, fall, and summer samples all overlapped significantly but the spring and summer samples were less variable than the fall samples (Supplemental Figure S2C).

**Figure 2:**
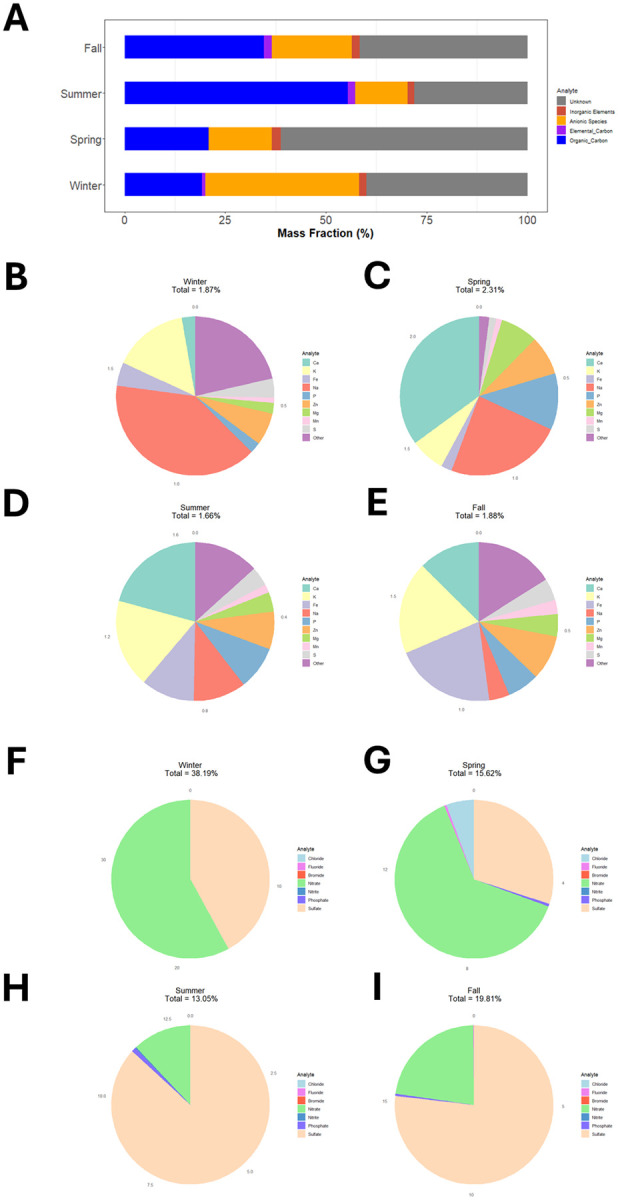
Chemical Composition of Particulate Matter by Collection Season. A) Percent mass fraction of major chemical classes by particle collection season. Pie charts describing proportion of B) inorganic elements and C) anionic species by particle collection season.

The chemical composition of PM_2.5_ samples selected for toxicology experiments demonstrated clear variability between chemical categories (Fig. 2A). Of all the samples, the combined inorganic element mass fraction was largest in the sample from Spring. Ca, K, Fe, Na, P, Zn, Mg, Mn, and S combined made up a large fraction of the inorganic component of the four samples (Fig. 2B). Compared to the other samples, the Winter PM sample, however, contained a large fraction of Al and the largest fraction of Cu. Interestingly, Si made up an appreciable fraction of the Fall and Summer PM samples, but not the Spring or Winter samples. The Spring PM sample had the largest mass fraction of Ca, Mg, and P of all the samples. The largest mass fraction of anionic components was measured in the Winter PM sample (Fig. 2C). The Summer and Fall PM samples contained much more sulfate than any other anion, followed by nitrate and a small fraction of phosphate. The Winter and Spring samples in contrast contained more nitrate than sulfate. Additionally, the largest mass fractions of chloride and fluoride in any of these four samples was measured in the Spring PM sample. Chemical component concentrations and mass fractions in these four PM samples are presented in Table 2.

### Seasonal PM exposure induces changes in the transcriptome

**Figure 3:**
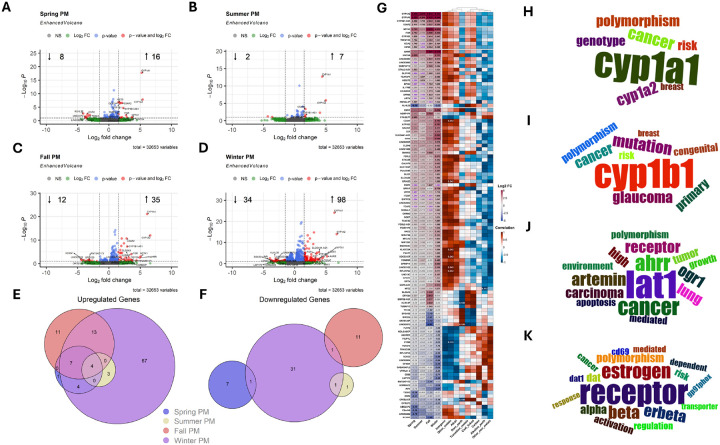
Particulate Matter Induces Seasonal Specific Gene Expression Patterns Two Hours Post Exposure. A-D) Volcano plots demonstrating significantly differentially expressed genes (DEGs) following exposure to indicated particles compared to vehicle. Euler plots presenting the number of unique and overlapping E) upregulated or F) downregulated DEGs between treatment groups. G) Heatmap presenting (left) log_2_ fold change of DEGs or (right) correlation values between PM chemical component mass fractions and resulting log_2_ fold changes clustered according to the number of groups demonstrating significant differential expression. Values within cells represents (left) log_2_ fold change values compared to vehicle exposure or (right) correlation p-values. Black text represents q value < 0.1 and absolute log_2_ fold change values ≥ 1.5. Purple text represents q value < 0.1 and absolute log_2_ fold change values ≤ 1.5. Grey text represents q value ≥ 0.1. Word clouds demonstrating major biological themes impacted by DEGs shared by H) four, I) three, J) two, K) or one exposure group.

Relative to the no particle control, exposure to seasonal PM induced unequal effects on the HNEC transcriptome at the 2 h timepoint (Fig. 3). Winter PM induced the highest number of DEGs, followed by Fall, Spring, and finally Summer PM (Fig. 3A–D). Euler plots indicate that the upregulated DEGs largely overlap (Fig. 3E); however, December PM demonstrates the greatest number of unique upregulated and downregulated DEGs (Fig. 3E/F). Importantly, four genes (*CYP1A1, CYP1A2, CYP1B1-AS1*, and *C5AR2*) demonstrated significant upregulation in all four exposure groups (Fig. 3G). Upregulation of *C5AR2* demonstrates modification of complement activity, suggesting PM may prime HNEC cells for complement-mediated innate immunity while upregulation of two major cytochrome P450 genes as well as the antisense RNA targeting of *CYP1B1* suggests each PM was capable of modifying xenobiotic metabolism. While only four genes demonstrated significant upregulation in all four exposure groups, the majority of DEGs induced by Spring, Summer, and Fall PM overlapped with Winter PM. Interestingly, *CYP1B1* demonstrated significant upregulation in all but Summer PM exposed groups (Supplemental File SF2), further demonstrating the impact of PM on initiating xenobiotic metabolism processes. In contrast, downregulated DEGs were largely unique to each PM (Fig. 3F); however, the total number of downregulated DEGs was notably lower than the total number of upregulated genes (53 versus 110). Importantly, few genes demonstrated significant differential expression at 24 hours post exposure (Supplemental Figure S3). While Fall PM demonstrated the greatest number, a total of only six DEGs were detected (Supplemental Figure S3C), suggesting PM has a negligible impact on HNEC gene expression profiles by 24-hours post exposure.

When considering the biological themes impacted within and between exposure seasons, major xenobiotic metabolism activity demonstrates the greatest impact. Specifically, CYP1A1 and to a lesser extent CYP1A2, driven by upregulation of their respective genes, demonstrated the greatest impact in each exposure group (Fig. 3H). Further, CYP1B1, driven by upregulation of *CYP1B1* demonstrated the greatest impact of genes demonstrating significant differential expression in Winter, Spring, and Fall PM exposed samples (Fig. 3I). Interestingly, while Summer PM exposed samples did not demonstrate significant differential expression according to our cutoffs of q-value < 0.1 and absolute log_2_ fold change of 1.5, Summer PM did demonstrate a q-value < 0.1 and log_2_ fold change of 1.254 (Fig. 3G), further suggesting every PM induced broad xenobiotic metabolism activity, with the degree of activity being season-dependent. Next, the L-amino acid transmembrane transporter Lat1 demonstrated the greatest thematic enrichment (Fig. 3J) due to the upregulation of its corresponding gene *SLC7A5*, however, the aryl hydrocarbon receptor (AHRR) also demonstrated enrichment due to the upregulation of its corresponding gene *AHRR* in Winter and Fall PM exposed samples (Fig. 3G) further cementing the enrichment of xenobiotic metabolism activity. Finally, receptor expression patterns were enriched within individual exposure groups (Fig. 3K), particularly estrogen receptor beta (*ESR2*) and G protein-coupled estrogen receptor 1 (*GPER1*) which demonstrated downregulation in Spring PM exposed samples (Fig. 3G).

The correlation between PM mass fractions and resulting fold change gene expression demonstrated that inorganic species, other metals (Al27, Ge72, Sn118, Tl205, Bi209), and alkali metals primarily demonstrated strongly positive while alkaline earth, carbon, and other non-metals (Si28, P31, S32) demonstrated strongly negative correlation coefficients with the exception of *CYBB* and *TNSF4*, which demonstrated the opposite trends (Fig. 3G). Importantly, despite demonstrating similar correlation trends to each other, coal-linked elements and transition metals demonstrated a mixture of positive and negative correlation patterns. However, despite frequently demonstrating strong correlations, few rose to the level of statistical significance (Fig. 3G).

### Seasonal PM induced differential expression of genes in response to infection

**Figure 4:**
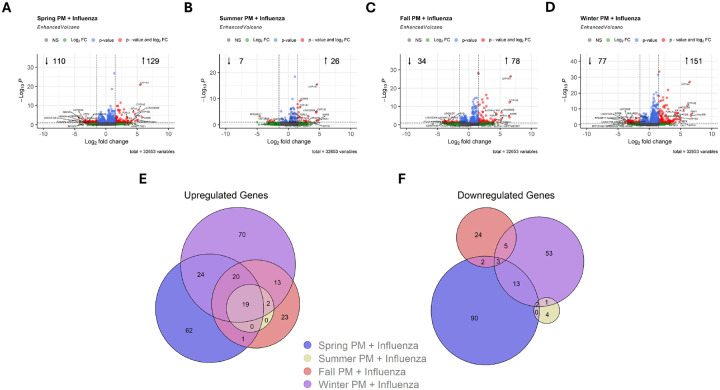
Particulate Matter and Influenza Co-Exposure Induces Unique Gene Expression Patterns Two Hours Post Exposure. A-C) Volcano plots demonstrating significantly differentially expressed genes (DEGs) following co-exposure to indicated particles and influenza compared to vehicle exposed, influenza infected controls. Euler plots presenting the number of unique and overlapping D) upregulated or E) downregulated DEGs between treatment groups.

Among infected cultures, exposure to seasonal PM did not induce differential expression of influenza H1N1 genes (Supplemental Table ST1). In contrast to PM exposure alone, the greatest number of DEGs following co-exposure to virus and PM were induced by Spring PM, followed by Winter, Fall, and finally Summer PM (Fig. 4A–D). 19 genes demonstrated shared upregulation while no genes shared downregulation in each exposure group (Fig. 4E/4F). While a substantial subset of downregulated genes were shared between at least two PM exposure group, the majority were unique to a single group (Fig. 4F), suggesting season-dependent interactions with influenza virus. When considering the impact of individual versus combined treatments at 2 h p.i., the majority of differentially expressed genes were not shared between individual and combined exposures (Supplemental Figure S4). Specifically, 23/96 genes which were differentially expressed by Winter PM were also differentially expressed in samples exposed to both PM and influenza (Supplemental Figure S4A–B). In contrast, no DEGs were shared between samples exposed to influenza alone when compared to samples exposed to both PM and virus (Supplemental Figure S4C–D). These findings suggest that the interaction between PM exposure and viral infection induces largely unique rather than additive gene expression changes. Interestingly, at 24 h p.i., there were few differentially expressed genes (Supplemental Figure S5).

**Figure 5:**
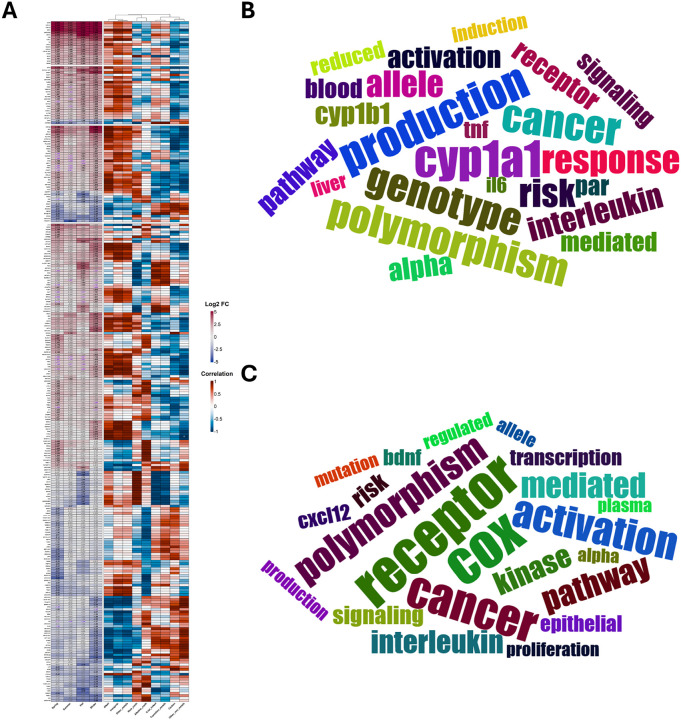
Spring and Winter Co-Exposure Induce Greater Responses following Co-Exposure at Two Hours Post Infection. A) Heatmap presenting (left) log_2_ fold change of DEGs or (right) correlation values between PM mass fractions and resulting log_2_ fold changes clustered according to the number of groups demonstrating significant differential expression. Values within cells represent (left) log_2_ fold change values compared to vehicle exposure or (right) correlation p-values. Black text represents q value < 0.1 and absolute log_2_ fold change values ≥ 1.5. Purple text represents q value < 0.1 and absolute log_2_ fold change values ≤ 1.5. Grey text represents q value ≥ 0.1. Word clouds demonstrating major biological themes impacted by DEGs shared by B) all exposure groups or observed following C) Winter and/or Spring co-exposure.

In agreement with PM exposure alone, *CYP1A1, CYP1A2, CYP1B1-AS1*, and *C5AR2* demonstrated upregulation in each co-exposure group compared to vehicle exposed, influenza infected samples; further, *CYP1B1* and *IL6* also demonstrated upregulation in each group (Fig. 5A) leading to biological term enrichment for CYP1A1, CYP1B1, and Interleukin (Fig. 5B). Interestingly, the number of DEGs we observed by seasonal PM sample mirrored the natural prevalence of influenza infection in humans which generally occur between November to March in the northern temperate zone^36–37^. As such, we focused further assessment on biological term enrichment in Winter or Spring PM co-exposed samples. Modification of cyclooxygenase (COX) was observed due to the shared upregulation of the gene encoding cyclooxygenase (*PTGS2*) while the term receptor demonstrated enrichment due to differential expression of numerous cell surface receptors including the complement C5a receptor 2 (*C5AR2*) and interleukin 17 receptor B (*IL17RB*) (Fig. 5A/5C). The term Interleukin also demonstrated enrichment due to shared upregulation of *IL6* as well as upregulation of *IL11* and *IL24* in Spring and Winter PM co-exposed samples, respectively (Fig. 5A/5C). Further, the term kinase demonstrated enrichment due to a wide range of changes in expression of kinases (Fig. 5C), however, this change was primarily seen following Spring PM co-exposure as demonstrated by unique upregulation of *CAMK2B* and *STK32B* and unique downregulation of *LRRK2, MAPK4*, and *PIK3CG* (Fig. 5A). However, *FGR* demonstrated upregulation following each PM exposure with Winter > Fall > Spring > Summer (Fig. 5A). Finally, the term CXCL12 demonstrated enrichment due to the upregulation of *CXCL12* exclusively in Spring PM co-exposed samples (Fig. 5A/C).

Importantly, in contrast to PM exposure alone, mass fractions of each PM component class demonstrated greater variability in measured correlation coefficients with resulting log_2_ fold changes in expression. However, as with PM exposure alone (Fig. 3H), similar patterns could be detected between classes of PM components. Specifically, as seen in PM exposure alone, the pair of Carbon components and other nonmetals, the pair of coal-linked and transition elements, and the trio of alkali elements, inorganic species, and other metals demonstrated linked correlation patterns for the majority of differentially expressed genes (Fig. 5A).

### Seasonal PM induces differential biomarker release

**Figure 6:**
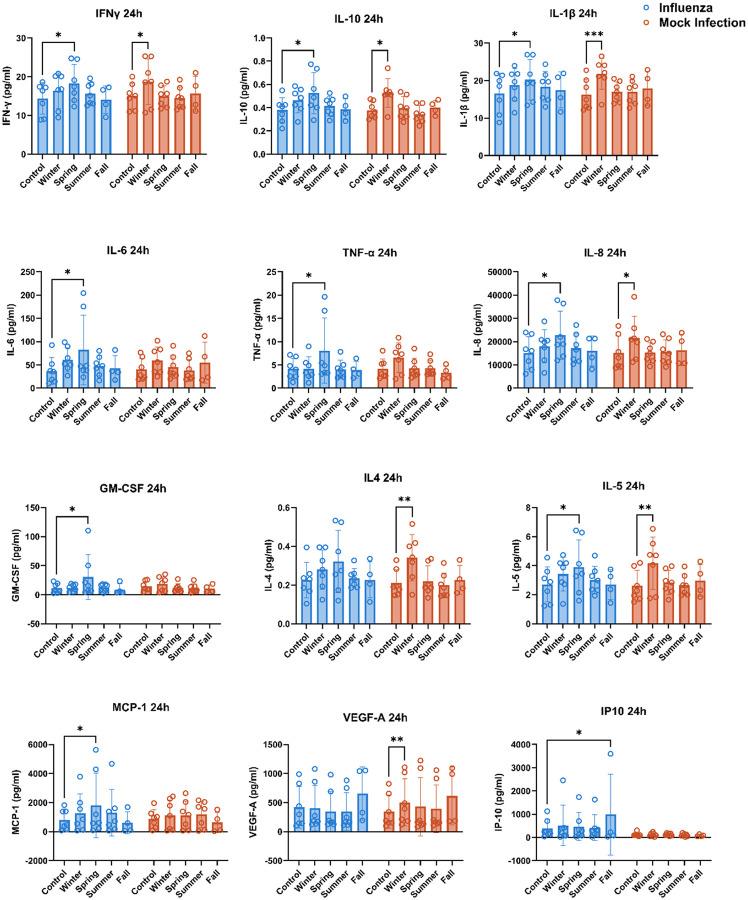
Biomarker Release Demonstrates Seasonal PM Specific Release. At 24 h p.i., basolateral supernatants were collected and levels of mediators related to inflammation, antiviral response, and immune cell recruitment were measured. A mixed effects model was used for statistical analysis using donor as a random variable and Dunnett’s post hoc test was used to test for differences between groups, * p ≤ 0.05, ** p ≤ 0.01.

Levels of basolaterally-released cytokines and chemokines related to viral infection response were measured at 24 h and biomarkers whose expression was statistically significantly affected by seasonal PM exposure are shown in Fig. 6. There were no statistically significant differences in biomarker release between males and females, thus data shown are sex aggregated. In the Influenza infection group, the Spring PM exposure induced elevated expression of IFN-γ, IL-1β, IL-6, IL-8, TNF-α, IL-10, MCP-1, and GM-CSF relative to the control exposure. In the mock infection group, however, the Winter PM exposure induced elevated IFN-γ, IL1-β, IL-8, IL-4, IL-5, IL-10, and VEGF-A at the 24 h timepoint. There were no statistically significant effects of Winter PM exposure in the influenza viral infection group, and likewise with Spring PM in the mock infection group.

## Discussion

In this study, we sought to determine how differences in chemical composition of PM across seasons contribute to different biological effects and modify antiviral host defense responses in the airway epithelium. We found that components of PM_2.5_ in Xinxiang, China vary seasonally, with organic components making up a larger mass fraction in warmer months and anionic components highest in winter. The inorganic mass fraction was relatively similar across all samples but varied in composition and variety of predominant metals. We evaluated the effects of infection with influenza (H1N1)pdm09, exposure to seasonal PM samples, or combination of both exposure and infection on the transcriptome in HNECs. Viral infection alone induced weak transcriptomic changes at 2h post infection (p.i.) before robust and unique changes at 24h p.i.. Short term exposure to PM alone again induced weak and transient transcriptomic changes with the most differential gene expression occurring in Winter PM exposed samples. Finally, exposure to PM prior to infection with virus induced gene expression patterns distinct from individual exposures or infection responses, suggesting the interactions between PM exposures and influenza infection is not simply additive, but rather induce unique responses that vary with seasonality of the PM samples.

While the same infection protocol was used across the different experimental groups, distinct gene expression patterns were observed in samples 2h and 24h post influenza infection (Fig. 1). Transcriptomic changes due to early influenza infection measured at 2h p.i. demonstrated overall limited DEG numbers (Fig. 1A). Further, when considering individual genes, limited links to viral infection were detected. Specifically, significant upregulation of *ST3GAL1-DT*, a divergent transcript of the influenza (H1N1)pdm09 entry receptor^38^, as well as *MALT1-AS1*, antisense RNA for MALT1, a potent activator of NF-κB and regulator of immunity^39–40^ was observed. In contrast, a much larger degree of DEGs were detected at 24h p.i. compared to mock infection (Fig. 1B) or compared to 2 h p.i. (Fig. 1C). Surprisingly, only one gene, novel transcript ENSG00000259539.1, demonstrated overlap between 2h and 24h post infection (Fig. 1D), demonstrating distinct early and late phase responses to viral infection. In further support of this notion, differential expression analysis of 24h p.i, samples utilizing 2h p.i. as the reference group demonstrated a large degree of overlap with 24h p.i. samples utilizing uninfected controls as reference with 213/260 DEGs being shared (Fig. 1D/E). One additional gene, *PILRA*, demonstrated differential expression in each group, however, 2 h p.i. samples demonstrated upregulation while 24 h p.i. samples compared to mock infection or 2 h p.i. demonstrated downregulation (Supplemental File SF1). While *PILRA* is mainly expressed in myeloid cells, PILAα plays a major role in immunosuppressive signaling which has been demonstrated to play a key role in the regulation of *S. aureus* induced pneumonia^41^.

Since infections with influenza virus generally occur between winter (November/December) and early spring (March) in northern temperate zones^36–37^, we aimed to determine whether seasonal variability in ambient pollution affected the interaction between PM and influenza. In line with this pattern, exposure to seasonal PM prior to influenza infections induced additional DEGs following a pattern of Spring > Winter > > Fall > > Summer (Fig. 4A–D). Importantly, log_2_ fold change values of individual linked genes including *AHRR, CYP1A1, CYP1A2, CYP1B1, IL6, UGT1A3*, and *UGT1A4* demonstrate similar expression levels across seasonal PMs (Fig. 3G/5A), suggesting universal effects on xenobiotic metabolism due to PM exposure with or without subsequent infection with influenza. However, as demonstrated by total and absolute increases in DEGs compared to influenza or PM exposure alone, exposure to Spring or Winter PM prior to infection with influenza demonstrated the greatest change in DEGs.

Importantly, biological processes predicted to be impacted by PM exposures prior to influenza infection demonstrated a mixture of shared and unique responses with unique seasonal-specific responses. Specifically, cyclooxygenase (COX) enrichment was observed in samples exposed to Winter and Spring PM prior to influenza infection due to shared upregulation of *PTGS2* (Fig. 5A/5C). While PTGS2 activity has previously been linked to influenza infection^42–43^ and PM exposure^44–45^, *PTGS2* upregulation was only detected in samples exposed to Spring and Winter PM prior to influenza infection (Fig. 5A). Additionally, upregulation of complement C5a receptor 2 (*C5AR2*) was observed following each PM exposure and *IL17RB* demonstrated upregulation following Winter PM exposure (Fig. 3A) which have been linked to increased pathogenic responses due to the link between Th17^46–47^ and complement activation^48–49^ in influenza patients with severe complications. Finally, expression of interleukins in general and *CXCL12* specifically demonstrated infection and PM-specific patterns characterized by unique upregulation of *IL24* following Winter PM and *IL11* and *CXCL12* upregulation following Spring PM co-exposure. While *IL24* expression has been shown following PM exposure^50–51^ and demonstrates antiviral activity^52–53^, neither PM nor influenza alone induced *IL24* at either 2 or 24 hours (Fig. 1), suggesting potentially elevated antiviral activity through IL-24-mediated priming through mechanisms such as TLR3-mediated apoptosis^53^. While *IL11* has demonstrated upregulation during bacterial pneumonia^54^, its role in pulmonary immunity is likely modest due to its limited role in neutrophil recruitment and bacterial clearance^55^. However, *IL11* has also demonstrated upregulation in response to viral infection^56–57^ and silica exposure^58^, suggesting it may be induced by a wide range of inflammatory agents. Finally, CXCL12 has been linked to progenitor cell and leukocyte recruitment into the lung during inflammation with a particular role in neutrophil recruitment^59^. Further, CXCL12 plasma levels have been linked to severe COVID-19^60–61^ while elevated BAL levels have been demonstrated in severe COVID-19 but not influenza^62^. Importantly, Spring PM exposure prior to influenza infection increased release of several cytokines as measured at 24 h p.i. which are indicative of cytokine storm in influenza infection: IL-6, IL-8, IL-10, and MCP-1^63–64^ (Fig. 6). Spring PM induced greater pro-inflammatory mediator release than other exposures in the infected cultures, while Winter PM induced the highest mediator release in mock-infected cultures. While inflammation is part of a normal response to infection and is necessary for recruitment of innate immune cells to quell the spread of infection, excessive inflammation in respiratory viral infection, termed cytokine storm, is associated with increased pathogenesis, intensive care unit admission, and mortality^63–66^. Together, these findings suggest that PM exposure prior to influenza infection induces an interactive effect which is not simply a combination of the effects induced by influenza or PM exposure alone. Overall, these data indicate that seasonal variability in the effects of ambient pollution on influenza-induced inflammation potentially has the worst outcome in PM collected during Winter and Spring, which are also peak seasons for influenza infections.

The average ambient PM_2.5_ concentration in Xinxiang during 2023 was 51.1 μg/m^3^ which is 10 times higher than the current World Health Organization guideline^5^. Substantial evidence has shown that PM_2.5_ concentration and chemical composition in China vary with season and are largely affected by seasonal centralized heating fueled by coal combustion^23^. We report larger mass fractions of nitrate in Winter and Spring PM_2.5_ compared to Summer and Fall. Coal combustion has been found to contribute to higher levels of nitrate found in wintertime Beijing PM^67^. Across the PM samples collected from November – March, organic carbon contributed a lower mass fraction compared to the samples from warmer months, June – October. This is in line with other findings, indicating carbonaceous compounds from biomass burning and biogenic secondary organic aerosols contribute a larger mass fraction of PM in warmer months^23, 68^. Additionally, we found that three elements associated with coal combustion, Pb, As, and Se^69^ were at their highest ambient concentrations in the Fall and Winter PM samples, though these elements did not necessarily have the highest mass fractions in these samples due to the differences in ambient PM concentration on the four sampling days.

Exposure to PM from each season induced activation of aryl hydrocarbon receptor (AHR) signaling^70^, evidenced by upregulation of *CYP1A1, CYP1A2*, and *CYP1B1-AS1* following each exposure, upregulation of *CYP1B1* following each exposure except Summer PM, while Winter and Fall PM induced upregulation of *SLC7A5* and *AHRR* (Supplemental File SF2). Polycyclic aromatic hydrocarbons (PAHs) found in PM are known to activate the AHR and result in upregulation of these phase I metabolism cytochrome P450 enzymes^71^. Though PAHs were not measured explicitly, upregulation of AHR and other metabolic pathways suggests PAHs were present in our PM samples. Three studies in Beijing concluded PAHs associated with PM_2.5_ were 10–14 times higher in the heating season than non-heating season^72–74^. Coal combustion and vehicular emissions were determined to be the main source contributors of PM-associated PAHs.

There are several drawbacks to our study which must be considered when applying our findings to human health. First, our use of 22 μg/cm^2^ PM for 2 hours represents a super-physiological dose over a relatively short period absent rare conditions or occupational exposures^102–104^. However, mathematical modeling has demonstrated that hot spots within the airway can be exposed to hundreds or thousands of times more PM than surrounding tissue^105–108^ while other studies have utilized similar dosages to simulate chronic *in vivo* exposures in short term *in vitro* studies^28, 109–111^. Additionally, although real-world PM samples from different times of year were utilized, these samples were not necessarily representative of chemical profiles from their entire respective seasons. Specifically, Fall displayed the largest amount of variability between samples, likely reflecting the fact that samples were collected in both 2021 and 2022. While aggregate PM samples from multiple sampling days within each season could provide more accurate seasonal-specific chemical composition and more representative biological responses, it is unclear whether a truly ‘representative’ composition exists or whether this composition would be representative of a given season between years. Finally, our use of particles isolated from ambient air does not include gaseous toxicants such as ozone which have demonstrated clear impacts on inflammatory responses^112–113^ and influenza infection^114^. As such, future work is required to integrate the impact of gas- and particulate phase components of ambient air pollution on influenza infectivity and host responses in epithelial cells.

## Conclusions

We observed that PM collected in Winter, Spring, and Fall in Xinxiang, China induced greater transcriptomic changes in HNECs compared to PM collected in Summer. Chemical composition analysis suggests a role of coal combustion products in driving biological responses. Furthermore, exposure to PM interacted with influenza (H1N1) pdm09 to enhance inflammatory signaling and transcriptomic changes compared to the additive effects of either stimulus alone. The greatest transcriptional changes occurred due to Winter, Spring, and Fall PM exposures, which coincides with influenza season in China. These findings indicate that certain PM components have adjuvant effects in the context of viral infections enhancing inflammatory signaling, which could worsen pathogenesis due to infection. These data further suggest that regulation of source contributors of PM may be more impactful for human health outcomes than regulation of overall PM levels.

## Supplementary Files

This is a list of supplementary files associated with this preprint. Click to download.

- SupplementalTableST1.docx
- SupplementalFileSF1.csv
- SupplementalFileSF2.csv
- SupplementalFileSF3.csv
- SupplementalFigureS11.png
- SupplementalFigureS21.png
- SupplementalFigureS31.png
- SupplementalFigureS41.png
- SupplementalFigureS5.png

## Figures and Tables

**Table 1:** Demographic information of HNEC donors.

	Male	Female
Total = 7	**n = 4**	**n = 3**
Mean Age ± SD:	**25.2 ± 3.6**	**27.5 ± 2.3**
Mean BMI ± SD:	**26.3 ± 2.5**	**22.8 ± 1.2**
Asian/Caucasian/Black:	**1/3/0**	**0/3/0**
Hispanic ethnicity:	**2**	**0**

**Table 2:** Air concentrations and mass fractions of chemical components in PM samples used for toxicology experiments.

	Air Concentration μg/m^3^				Mass Fraction (%)			
Chemical Component	Winter 12/22/2021	Spring 3/18/2022	Summer 6/11/2022	Fall 9/28/2022	Winter 12/22/2021	Spring 3/18/2022	Summer 6/11/2022	Fall 9/28/2022
	60.2 μg/m^3^	22.2 μg/m^3^	11.7 μg/m^3^	17.7 μg/m^3^				
Organic C	11.56	4.64	6.51	6.58	19.2	20.925	55.5	34.58
Elemental C	0.5	0	0.2	0.39	0.83	0	1.73	2.03
Li	1.85E-03	3.33E-04	1.08E-04	5.77E-04	3.06E-03	1.50E-03	9.21E-04	3.03E-03
Be	1.72E-05	8.42E-07	2.11E-07	4.72E-06	2.86E-05	3.80E-06	1.80E-06	2.48E-05
Rb	1.14E-03	2.25E-04	1.33E-04	3.57E-04	1.89E-03	1.02E-03	1.14E-03	1.87E-03
Sr	1.25E-04	6.21E-04	1.12E-04	1.09E-04	2.07E-04	2.80E-03	9.50E-04	5.73E-04
Y	2.95E-05	4.10E-07	5.06E-06	3.62E-06	4.91E-05	1.85E-06	4.31E-05	1.90E-05
Mo	1.22E-03	2.51E-04	3.59E-04	1.40E-03	2.03E-03	1.13E-03	3.06E-03	7.37E-03
Ag	7.32E-06	1.32E-06	1.97E-06	1.67E-05	1.22E-05	5.95E-06	1.68E-05	8.76E-05
Cd	1.82E-03	5.53E-04	1.13E-04	5.85E-04	3.02E-03	2.50E-03	9.61E-04	3.07E-03
Sn	1.38E-03	2.51E-04	5.47E-04	8.69E-04	2.30E-03	1.13E-03	4.67E-03	4.57E-03
Sb	8.20E-03	1.47E-03	4.30E-03	3.66E-03	1.36E-02	6.65E-03	3.67E-02	1.92E-02
Cs	3.99E-05	8.01E-05	0.00E + 00	1.33E-05	6.63E-05	3.61E-04	0.00E + 00	6.97E-05
Ba	5.46E-04	5.27E-04	1.39E-04	5.31E-04	9.06E-04	2.38E-03	1.19E-03	2.79E-03
La	2.64E-05	0.00E + 00	5.86E-06	8.10E-06	4.39E-05	0.00E + 00	5.00E-05	4.26E-05
Ce	5.23E-05	6.69E-05	1.62E-05	1.91E-05	8.68E-05	3.02E-04	1.39E-04	1.00E-04
Nd	2.82E-05	0.00E + 00	4.17E-06	4.21E-06	4.68E-05	0.00E + 00	3.56E-05	2.21E-05
Sm	1.20E-05	0.00E + 00	8.56E-07	8.57E-07	1.99E-05	0.00E + 00	7.30E-06	4.50E-06
Gd	1.26E-05	3.32E-07	1.51E-06	1.62E-06	2.09E-05	1.50E-06	1.29E-05	8.50E-06
Tb	6.65E-06	0.00E + 00	1.64E-07	1.05E-07	1.11E-05	0.00E + 00	1.40E-06	5.50E-07
Dy	9.30E-06	0.00E + 00	7.57E-07	2.38E-07	1.55E-05	0.00E + 00	6.45E-06	1.25E-06
W	0.00E + 00	0.00E + 00	0.00E + 00	5.71E-05	0.00E + 00	0.00E + 00	0.00E + 00	3.00E-04
Tl	3.09E-04	1.10E-04	4.81E-05	1.46E-04	5.12E-04	4.97E-04	4.10E-04	7.65E-04
Pb	7.83E-03	1.53E-03	1.15E-03	7.55E-03	1.30E-02	6.89E-03	9.81E-03	3.96E-02
Bi	8.95E-04	2.81E-05	9.98E-05	6.04E-04	1.49E-03	1.27E-04	8.51E-04	3.17E-03
Na	4.48E-01	1.22E-01	2.10E-02	1.50E-02	7.44E-01	5.49E-01	1.79E-01	7.90E-02
Mg	2.44E-02	3.92E-02	7.74E-03	1.64E-02	4.04E-02	1.77E-01	6.60E-02	8.62E-02
Al	1.72E-01	0.00E + 00	0.00E + 00	0.00E + 00	2.85E-01	0.00E + 00	0.00E + 00	0.00E + 00
Si	0.00E + 00	0.00E + 00	1.33E-02	2.24E-02	0.00E + 00	0.00E + 00	1.14E-01	1.18E-01
P	2.48E-02	5.89E-02	1.73E-02	2.34E-02	4.13E-02	2.66E-01	1.48E-01	1.23E-01
S	4.40E-02	7.34E-03	7.68E-03	1.62E-02	7.31E-02	3.31E-02	6.54E-02	8.49E-02
Ca	3.10E-02	1.79E-01	4.03E-02	4.51E-02	5.16E-02	8.09E-01	3.44E-01	2.37E-01
Ti	4.27E-04	6.27E-05	3.22E-04	9.10E-04	7.09E-04	2.83E-04	2.74E-03	4.78E-03
V	3.42E-04	1.26E-04	2.27E-04	1.56E-04	5.67E-04	5.69E-04	1.94E-03	8.21E-04
Cr	1.28E-03	4.26E-04	2.61E-04	1.24E-03	2.12E-03	1.92E-03	2.23E-03	6.49E-03
Mn	1.22E-02	6.17E-03	3.24E-03	1.03E-02	2.03E-02	2.78E-02	2.77E-02	5.39E-02
Fe	5.35E-02	1.13E-02	2.13E-02	7.40E-02	8.89E-02	5.11E-02	1.81E-01	3.89E-01
Co	3.89E-05	1.23E-05	1.03E-03	4.59E-05	6.46E-05	5.53E-05	8.73E-03	2.41E-04
Ni	7.51E-05	8.23E-04	3.14E-04	6.49E-04	1.25E-04	3.72E-03	2.68E-03	3.41E-03
Cu	2.48E-02	7.92E-04	1.49E-03	4.24E-03	4.11E-02	3.58E-03	1.27E-02	2.23E-02
Zn	7.49E-02	4.09E-02	1.49E-02	3.29E-02	1.24E-01	1.85E-01	1.27E-01	1.73E-01
K	1.74E-01	3.62E-02	3.51E-02	6.78E-02	2.89E-01	1.63E-01	2.99E-01	3.56E-01
Ge	2.53E-04	3.42E-05	3.00E-05	1.24E-04	4.20E-04	1.54E-04	2.56E-04	6.51E-04
As	1.46E-02	1.78E-03	7.38E-04	7.77E-03	2.42E-02	8.02E-03	6.29E-03	4.08E-02
Se	4.44E-03	7.45E-04	1.17E-03	4.16E-03	7.37E-03	3.36E-03	9.93E-03	2.18E-02
Fluoride	0.006	0.016	0	0.006	0.01	0.072	0	0.031
Chloride	0	0.197	0	0	0	0.887	0	0
Nitrite	0	0	0.004	0	0	0	0.033	0
bromide	0	0	0	0	0	0	0	0
Sulfate	9.666	1.04	1.329	2.899	16.051	4.691	11.329	15.221
Nitrate	13.324	2.191	0.184	0.853	22.125	9.888	1.573	4.476
Phosphate	0	0.019	0.013	0.016	0	0.086	0.115	0.085

## Data Availability

All R codes associated with this manuscript can be found at: [private link for reviewers] https://dataverse.unc.edu/previewurl.xhtml?token=3a022d36-a776-4cdc-ac86-5d50a2be3326. All raw data and supplemental files associated with this manuscript can be found at: [private link for reviewers] https://dataverse.unc.edu/previewurl.xhtml?token=3a022d36-a776-4cdc-ac86-5d50a2be3326.
